# Improving clinical reasoning competency and communication skills using virtual simulation-based learning focused on a pathophysiological approach in Korea: a quasi-experimental study

**DOI:** 10.7586/jkbns.24.026

**Published:** 2024-11-19

**Authors:** Sung Hae Kim, Yoona Choi

**Affiliations:** 1 Department of Nursing, Tongmyong University, Busan, Korea; 2 Department of Nursing, Ulsan College, Ulsan, Korea

**Keywords:** Clinical reasoning, Communication, Simulation training, Pathology, Physiology

## Abstract

**Purpose:**

Clinical reasoning, which is based on an understanding of the pathophysiological mechanisms of diseases, is a core nursing competency that involves analyzing patient-related data and providing appropriate nursing practices. Simulation-based education is effective in improving clinical reasoning competencies and communication skills. This study evaluated the effectiveness of virtual simulation-based learning in improving the communication skills and clinical reasoning competencies of undergraduate nursing students.

**Methods:**

This study used a single-group pretest and posttest quasi-experimental design to evaluate the effectiveness of virtual simulation-based learning. Data were collected from June to September 2020. Thirty-seven nursing students in their third and fourth years of study who understood the purpose of this study were selected as participants. The collected data were analyzed using SPSS Statistics 25.0 and Winsteps 3.68.2.

**Results:**

The communication skills (t = −12.80, *p* < .001) and clinical reasoning competency (t = −4.67, *p* < .001) of the undergraduate nursing students who participated in the virtual simulation-based learning program improved significantly after participation. Additionally, a Rasch model analysis revealed that the overall clinical reasoning competency of undergraduate nursing students improved.

**Conclusion:**

Virtual simulation-based learning programs for nursing students should be developed and implemented.

## INTRODUCTION

Nurses must possess core competencies, including an understanding of the pathophysiological mechanisms of diseases, to effectively perform the nursing process, which involves a series of interconnected steps from subjective and objective data collection to nursing diagnosis, planning, implementation, and evaluation, to care for patients with complex and diverse clinical symptoms [1]. Clinical reasoning competency, based on an understanding of the pathophysiological mechanisms of diseases and effective communication skills, is essential for nurses in patient safety and evidence-based nursing practice [2-4]. In addition to understanding diseases, clinical reasoning competency and effective communication skills help them collect and analyze subjective and objective data to prioritize nursing interventions and make decisions to address patients’ health problems [4-6].

Clinical reasoning is defined as a complex cognitive process that uses formal, informal, and empirical thinking strategies to gather and analyze information about a subject, evaluate the significance of this information, and select alternatives [7]. Through a conceptual analysis, clinical reasoning was analyzed into nine attributes: cognition, information processing, analysis, metacognition, heuristics, deliberation, intuition, inference, and logic [7]. Clinical reasoning competency helps nurses collect subjective and objective data, analyze and organize data to identify specific problems, set priorities for solving health problems, and select problem-solving solutions [5,8]. Therefore, nurses can understand and analyze patients’ health problems through a pathophysiological knowledge of diseases with signs and symptoms, select important data, and enhance their clinical reasoning competency to provide appropriate nursing interventions according to priorities [6,9,10].

Clinical reasoning competency and communication skills are becoming increasingly important for healthcare professionals, including nurses [11,12]. Effective communication is an essential factor in patient–nurse interactions, and higher communication skills can enhance nurses’ problem-solving abilities [13]. As a pathophysiological approach to diseases, clinical reasoning competency and communication skills are emphasized as a core competency of nurses; interest in educational methods to improve clinical reasoning competency in undergraduate nursing education is increasing. For undergraduate nursing students, clinical practicums provide opportunities to integrate nursing knowledge with practical skills based on an understanding of the pathophysiology of diseases and identify the patient's disease manifestations, thereby enhancing their clinical reasoning competencies [14,15]. However, due to the recent emphasis on patient safety and patient rights in clinical settings, direct nursing practice for patient care among these students has been restricted [13,16]. Consequently, opportunities for experiential learning through diverse case studies have diminished.

Simulation-based training is applied to medical and nursing education as an alternative, providing a controlled and safe practice environment related to patient safety management. It is also effective in improving learners' clinical reasoning competency, problem-solving competency, and communication skills [10,17,18]. Nursing students can understand disease manifestations and develop their clinical reasoning competency, critical thinking skills, and communication skills through repeated learning in a safe environment using case study-based simulation education [19]. Simulation-based training enables nursing students to comprehend the complexity of clinical situations and to develop and refine their clinical reasoning skills through decision-making, critical reflection, and problem-solving [5,19]. Virtual simulation education can be motivated to learn by constructing actual clinical sites as virtual reality learning environments [2,20]. Furthermore, non-immersive 2D (e.g., screen-based simulation) simulation training can reduce learning cost, learners’ anxiety, and cognitive loading, compared to immersive 3D virtual simulations [21]. Additionally, 2D simulations are more suited to build fundamental knowledge. They allow learners to grasp theoretical concepts and basic procedures in a straightforward and cost-effective manner, making them ideal interventions [22]. In previous studies, virtual simulation education has also been reported to effectively promote clinical reasoning competency among nursing students [10].

The Rasch model is a method of estimating an individual’s ability value through test equalization based on the item response theory [23,24]. The Rasch model analysis method is useful for assessing changes in individual abilities, as it evaluates these changes by analyzing pretest and posttest data in the context of specific educational interventions [25]. Previous research has utilized Rasch model analysis to observe changes in undergraduate students' learning competencies over time [25,26]. This study aimed to verify the usefulness of virtual simulation-based learning for undergraduate nursing students, focusing on clinical reasoning competency and communication skills. Additionally, it analyzed the degree of improvement in clinical reasoning competency using the Rasch model.

## METHODS

### 1. Study design

This quasi-experimental study used a single-group pretest-posttest design to verify the effectiveness of virtual simulation-based learning for undergraduate nursing students.

### 2. Participants

The participants were third- and fourth-year undergraduate nursing students who understood the study’s purpose and voluntarily agreed to participate. The participants were recruited from the department of nursing at Tongmyong University in South Korea. The calculation of the sample size was based on a paired sample t–test using G Power 3.1.9 (α = .05, effect size = .5, 1−ß = .80), and the minimum number of samples was estimated to be 34. Thirty-seven nursing students participated in this study. The participants completed a pretest before the intervention and a posttest after the intervention.

### 3. Interventions

**Figure 1.**
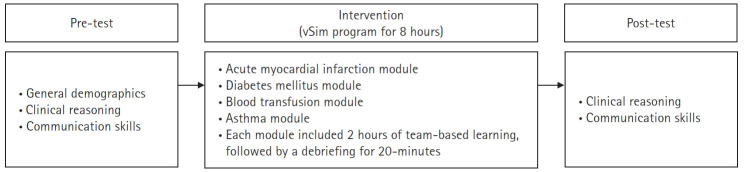
Study flow for virtual simulation-based learning program.

We used “vSim^®^ for Nursing - Nursing Medical-Surgical (Laerdal Medical, Norway)” as the virtual simulation-based learning program. The intervention procedures are presented in Figure 1. The vSim learning program used four modules and was structured around scenarios specific to each module. This includes learning activities to understand the pathophysiological mechanisms of each disease; assess fundamental patient health assessments based on disease manifestations; and practice nursing interventions such as vital signs measurement, oxygen therapy, transfusion management, blood glucose monitoring, and medication management. The program aims to improve nursing practice skills, including clinical reasoning and communication competencies for participants. Four modules were included: (a) acute myocardial infarction (MI), (b) diabetes mellitus, (c) transfusion, (d) asthma. Each module learning session was conducted based on scenarios, which consisted of five steps: suggested reading, pre-simulation quiz, vSim, post-simulation quiz, and guided reflection questions. In the “suggested reading” and “pre-simulation quizzes” steps, the instructor provided additional explanations of disease-specific pathophysiologic mechanisms and key nursing points beyond those provided in the vSim module. These sessions were conducted for 30 minutes as learning phases to learn the pathophysiological mechanisms of the disease through scenario-specific clinical symptoms and nursing diagnosis, and to develop nursing plan and evaluate nursing interventions. For example, the pathophysiological approach to MI was conducted by learning about the characteristics of myocardial changes in the event of MI, hemodynamic diagnostic tests such as CK-MB and troponin, understanding of myocardial injury and ST elevation MI (STEMI), and pathophysiological knowledge on the decreases of the preload and afterloads of the heart, as well as pain relief when morphine was administered. Following this, during the “vSim” and “post-simulation quiz” phases for 70 minutes, fundamental knowledge on pathophysiological mechanisms is applied to learn nursing interventions such as patient evaluation, O2 supply, drug administration according to patient condition changes, and patient education. In the “guided reflection questions” phase (debriefing), participants completed a reflection question sheet, followed by an instructor–learner discussion and feedback for 20 minutes of each module. Participants were provided a total of 8 hours of training, divided into 2 hours for each module. Even after the team's learning sessions were completed, individual login IDs and passwords were provided to allow participants to continue self-learning using “vSim for Nursing.”

### 4. Instruments

#### 1) Nurses clinical reasoning scale

The Nurses Clinical Reasoning Scale (NCRS) includes 15 items rated on a five-point Likert scale (1 = strongly disagree, 5 = strongly agree) [27]. The NCRS consists of items that inquire about patient assessment, identification of causal mechanisms related to health problems, understanding of pathophysiological mechanisms associated with symptoms and signs, and judgment of nursing priorities. We used the Korean version of the NCRS in this study [28]. Permission to use this instrument was obtained from the author. Higher scores indicated better clinical reasoning. Cronbach’s α was .94 for the original NCRS and .93 for the Korean version of the NCRS. In this study, Cronbach’s α was .94 for both the pretest and posttest.

#### 2) Communication skills scale

The Global Interpersonal Communication Competence Scale (GICC-15) includes 15 items rated on a five-point Likert scale (1 = strongly disagree to 5 = strongly agree) [29]. We used the Korean version of the GICC-15 [30]. Permission to use this instrument was obtained from the author. Cronbach’s α in Hur’s (2003) study was .72, and Cronbach’s α in this study was .89 (pretest) and .88 (posttest) [30].

### 5. Data collection

Pretest and posttest data were collected from the study participants. The participants completed self-reported questionnaires both pretest and posttest. The data were obtained from June to September 2020.

### 6. Data analysis

The data were analyzed using SPSS/WIN 25.0 (IBM Corp., Armonk, NY, USA), and Winsteps 3.68.2 software (Winsteps, Beaverton, OR, USA). Descriptive statistics (i.e., mean and standard deviation) and paired sample t-tests were used to summarize the participants' general characteristics and main outcomes. Rasch model analysis was conducted to calculate and verify item fit, item difficulty parameters, and mean square (MNSQ) values. Item fit was considered appropriate when the infit and outfit MNSQ values ranged from 0.4 to 1.6, and the corrected item-total correlation values were greater than 0.4 [23,31]. Item difficulty parameters were evaluated based on the distribution of item and person measurements on the item-person map. Higher values indicated items with greater difficulty parameters, while lower values indicated easier items.

### 7. Ethical considerations

This study was registered with clinicaltraials.gov (NCT06373172) and was approved by the Institutional Review Board (202005-HR-003). Students who voluntarily agreed to participate in the study responded to the questionnaire after providing informed consent. To adhere to ethical considerations, the research team assured students that there would be no consequences for non-participation, and that they could withdraw from the study at any time.

## RESULTS

### 1. Demographic characteristics

The general characteristics of the study participants are presented in Table 1. A total of 37 nursing students participated in this study. The mean age was 22.62 ± 3.15 years, and none of the participants had any experience in virtual simulation-based learning.

### 2. Intervention outcomes

Details of the changes in communication skills (t = −12.80, *p* < .001) and clinical reasoning competency (t = −4.67, *p* < .001) are presented in Table 2. There was a statistically significant increase in the post-intervention scores compared to the pre-scores.

### 3. Rasch analysis

**Figure 2.**
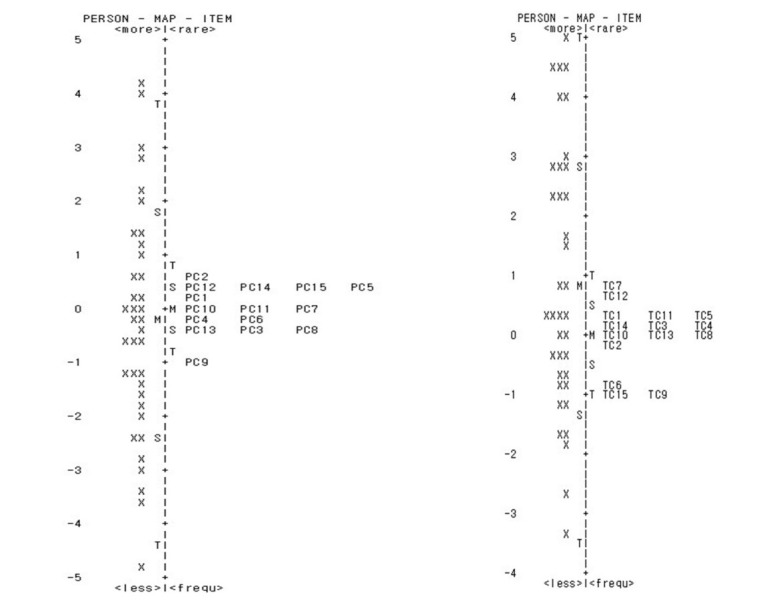
Item map comparing pretest and posttest scores for clinical reasoning in the virtual simulation-based learning program.

Regarding clinical reasoning competency, the infit MNSQ score ranged from 0.73 to 1.31, which satisfied the criteria for the goodness of fit of the item. As such, an improvement in clinical reasoning competency was observed (Table 3). The difficulty of each detailed item for measuring clinical reasoning competency was as follows: the easiest item for the pretest was nine, and the most difficult question was two. Contrarily, the easiest item for the posttest was nine, and the most difficult item was seven. Additionally, in item mapping, 10 participants (27.0%) in the pretest and 15 participants (40.5%) in the posttest who measured so high measure level in clinical reasoning (Figure 2).

## DISCUSSION

This study aimed to identify the effects of a virtual simulation-based learning program on nursing students. Virtual simulation-based learning improved nursing students' clinical reasoning competency in this study. The item mapping results also confirmed that the number of participants whose clinical reasoning competency was measured at a high level increased by 1.5 times compared to the pretest. This finding is consistent with that of a prior study [32]. Another study showed that clinical reasoning competency was higher in a virtual simulation-based learning group centered on the application of knowledge and practice [21]. Additionally, this study provided that virtual simulation-based learning activities emphasizing a pathophysiological approach to diseases can enhance participants’ clinical reasoning competency; this aids in accurately explaining the causal mechanisms related to patient health problems. The importance of clinical reasoning competency for nurses was originally discussed in the United States in 2010 [33]. According to the American Nurses Association, clinical reasoning should be taught as a framework for the application of the nursing curriculum, as it is defined as a competency required for integrated problem-solving in clinical situations [34]. Advances in information and communications technology made it possible to improve clinical reasoning in nursing using virtual patients in virtual clinical environments through simulation education [35]. Virtual simulations-based learning utilizes clinical scenarios to help learners achieve learning objectives, is highly customizable, and improves clinical decision-making skills [21,36]. Virtual simulation scenarios can be developed based on complex clinical situations and implemented in a secure virtual space. It is also helpful for students to challenge their clinical reasoning through individual feedback [35], which facilitates learning [37] and is effective in providing repetitive and structured learning.

The results showed that the communication skills of the nursing students who participated in the virtual simulation-based learning program improved after participation. A previous randomized controlled trial (RCT) that compared communication skills between face-to-face live simulation and computer-based virtual simulation found that communication skills increased in both live simulation and computer-based virtual reality simulation, and there was no difference in communication skills between both methods [38]. This suggests that a simulation-based learning program can contribute significantly to the improvement of communication skills. Additionally, virtual simulation-based learning is as effective in increasing communication skills as live simulation, which requires more preparation for training. Virtual simulation-based learning has also shown that communication skills can be fostered in nursing education through a variety of participants, including patients and healthcare workers [38,39]. Although live simulation learning is an effective learning method, it requires more effort in terms of cost, because it needs an expensive high-fidelity simulator, a variety of equipment and resources, and laboratory equipment for training. Recently, the number of programs that use virtual simulation-based learning in nursing education has increased [39]. Compared to live simulation learning, virtual simulation-based learning is more sustainable [38] and allows for anonymity, which is less likely to cause social anxiety and stress among students [40]. In this respect, it provides a safe environment for students and allows them to interact with a variety of patients, which helps improve their communication skills.

In this study, the Rasch model analysis was used to identify the changes in clinical reasoning competency between pretest and posttest. The raw scores, such as the total score by adding each item or the item group score, included measurement error [41]. Previous studies have shown that Rasch model analysis obtains more accurate research results than when using raw scores [42]. The Rasch model helps to estimate the individual’s competencies through test equalization [23]. Moreover, it has the advantage of being able to identify the changes in individual competencies by pretest and posttest [25]. In this study, the results of the Rasch model analysis showed that clinical reasoning competency improved in the posttest, especially when analyzed by item. In particular, the clinical reasoning competency pertaining to the items “I know the follow-up steps to take if the patient's condition does not improve” (Item 15); “I can explain the mechanism and development associated with the initial signs and symptoms when a patient’s health deteriorates” (Item 6); and “I can apply proper assessment skills to collect a patient's current health information” (Item 2) improved after the virtual simulation-based learning program. This study provided virtual simulation-based learning activities emphasizing a pathophysiological approach to diseases and established evidence for the positive effects of enhancing clinical reasoning competency. Virtual simulation-based learning can help students continue to reason through various possibilities and identify the next steps by showing them a patient's condition that may not continue to improve, rather than the expected outcome of applying theoretical knowledge based on a pathophysiological approach to diseases and nursing practice. These situations frequently occur in clinical settings; however, they are difficult to recreate using teaching methods other than simulations. Simulation education helps students identify multiple nursing interventions to improve patient outcomes and manage complex situations [43].

Recognizing patient deterioration is important for patient safety. After becoming nurses, nursing students are expected to address various patient emergencies. However, they may not experience patient deterioration during their nursing practicum [44]. Owing to their lack of experience with deteriorating patients, improving their clinical reasoning competency through simulation learning and experience with emergency situations is necessary. Recognizing the early signs and symptoms associated with patient deterioration is also important; it becomes more useful when integrated with the knowledge of pathophysiological mechanisms of diseases. Goldsworthy et al. (2021) found that students' recognition of a rapidly deteriorating patient improved through virtual simulation-based learning [44]. This is similar to the results in Item 6, “I can explain the mechanism and development associated with the initial signs and symptoms when a patient’s health deteriorates,” which improved in this study. Virtual simulation-based learning can contribute to improving nursing students' clinical reasoning competency for patient problem-solving by demonstrating various emergency situations through a variety of scenarios. Simulation learning also helps nursing students assess the various nursing needs of patients [21].

In this study, virtual simulation-based learning increased the score of Item 2, “I can apply proper assessment skills to collect a patient's current health information.” On-campus nursing education practice can be used to assess patients through role-play, but there are limitations in implementing the same environment as the actual patient's problem. Simulation learning using a high-fidelity simulator is helpful for patient assessment by setting up a nursing problem situation. However, it requires personnel, such as operators and various equipment, for proper training. In contrast, computer-based virtual simulations allow students to make necessary assessments using simple tools. A previous study [45] showed that virtual simulation-based learning contributes to improving patient assessment skills among nursing students. Virtual simulation-based learning has the advantage of helping students make necessary assessments through virtual situations, similar to reality, and learning repeatedly.

However, this study found that Item 7, “I can accurately prioritize and manage any identifiable patient problems,” was more difficult for nursing students on the posttest than on the pretest. During simulation learning, students simultaneously experience various patient needs in emergencies and are confused about which one to prioritize. The previous study found that prioritization in simulation learning was the most difficult for students [46]. Nursing education emphasizes the importance of rapid patient assessment and the prioritization of nursing diagnoses. While students may believe themselves to be theoretically familiar with prioritizing patient health problems before virtual simulation-based learning, when various problems occur simultaneously in a patient through virtual scenarios, solving them using integrated thinking is difficult. To address these issues and ensure proper prioritization among nursing students, ongoing education through virtual simulation-based learning, which allows for repeated learning, is necessary.

Although this study identified the effectiveness of virtual simulation-based learning for nursing students, it has limitations related to its design. This quasi-experimental study used only one group. Therefore, the findings of the present study should be interpreted with caution. Further studies are required to explore the effectiveness of virtual simulation-based learning using RCT. This study presents the potential carryover effect resulting from the time interval between the pretest and posttest measurements for two months. Knowledge or learning experiences gained during the pretest may have influenced responses in the posttest, thereby potentially impacting the validity of the study’s findings. Additionally, while this study attempted to utilize virtual simulation-based education with a pathophysiological approach to diseases, there were limitations in measuring the outcome variable related to fundamental pathophysiological knowledge level of major diseases such as MI, diabetes, and asthma. Future studies are recommended to consider this in their research design.

## CONCLUSION

This study verified the efficacy of a virtual simulation-based learning program for undergraduate nursing students. The results revealed that clinical reasoning competencies and communication skills improved following the virtual simulation-based learning program. While previous studies have examined the relationship between virtual simulation-based learning and these competencies, this study notably incorporates a pathophysiological approach tailored to specific diseases, signs and symptoms, nursing diagnosis, and nursing practice within the virtual simulation-based learning framework. This approach enhanced undergraduate nursing students' understanding of clinical manifestations, disease mechanisms, and progression stages, thereby enabling them to recognize patient deterioration and apply appropriate assessment skills and nursing intervention. This strengthened their clinical reasoning competency and communication skills. Given the important role of clinical reasoning competencies and communication skills in solving patient health problems, it is possible to explore ways to utilize virtual simulation-based learning programs to enhance clinical competencies by integrating underlying pathophysiological knowledge.

## Figures and Tables

**Table 1.** General Characteristics of the Participants (N = 37)

Characteristics	Categories	n (%)/ M ± SD
Sex	Men	7 (18.9)
	Women	30 (81.1)
Age (yr)		22.62 ± 3.15
Year of study	Third	10 (27.0)
	Fourth	27 (73.0)
Experience of high-fidelity simulation education	Yes	28 (75.7)
	No	9 (24.3)
Experience of virtual simulation education	Yes	0 (0.0)
	No	37 (100)

M = Mean; SD = Standard deviation.

**Table 2.** Comparison of Outcomes between the Pretest and Posttest (N = 37)

Variables	Pre	Post	t	*p*
	M ± SD			
Communication skills	49.73 ± 8.77	61.05 ± 7.74	−12.80	< .001
Clinical reasoning	57.73 ± 5.42	62.24 ± 5.79	−4.67	< .001

M = Mean; SD = Standard deviation.

**Table 3.** Item Analysis of the Rasch Model Analysis for Clinical Reasoning Competency (N = 37)

Item	Measure	Infit	Outfit	Corrected item-total correlation
		MNSQ		
1	.20	1.03	1.07	.67
2	.52	1.21	1.14	.70
3	−.44	1.21	1.18	.63
4	−.13	1.01	0.95	.74
5	.45	1.31	1.30	.67
6	−.24	0.80	0.78	.81
7	.04	0.83	0.85	.77
8	−.32	0.75	0.79	.82
9	−.91	0.73	0.68	.81
10	.00	0.96	0.98	.72
11	−.04	0.97	1.04	.68
12	.35	0.91	0.89	.78
13	−.41	1.13	1.12	.73
14	.46	1.02	1.11	.71
15	.46	0.78	0.84	.79

MNSQ = Mean square.

## Data Availability

Please contact the corresponding author for data availability.
